# Supplementary material for: Hydroboration of Terminal Alkenes and trans‐1,2‐Diboration of Terminal Alkynes Catalyzed by a Manganese(I) Alkyl Complex
Source: Angew Chem Weinheim Bergstr Ger. 2021 Oct 13;133(46):24693–7. doi: 10.1002/ange.202110736 (PMC10947181; doi:10.1002/ange.202110736)

checkCIF/PLATON report

Structure factors have been supplied for datablock(s) Mndippe_borane

THIS REPORT IS FOR GUIDANCE ONLY. IF USED AS PART OF A REVIEW PROCEDURE FOR PUBLICATION, IT SHOULD NOT REPLACE THE EXPERTISE OF AN EXPERIENCED CRYSTALLOGRAPHIC REFEREE.

No syntax errors found. CIF dictionary Interpreting this report

Datablock: Mndippe_borane

```
Bond precision:  C-C = 0.0032 A          Wavelength=0.71073

Cell:           a=7.8628(5)       b=12.3332(7)     c=14.5358(9)
                alpha=80.129(2)   beta=78.882(2)   gamma=75.362(2)
Temperature:    100 K

                 Calculated                 Reported
Volume           1327.02(14)                1327.02(14)
Space group      P -1                       P -1
Hall group       -P 1                       -P 1
Moiety formula   C22 H46 B Mn O4 P2         ?
Sum formula      C22 H46 B Mn O4 P2         C22 H46 B Mn O4 P2
Mr               502.28                     502.28
Dx,g cm-3        1.257                      1.257
Z                2                          2
Mu (mm-1)        0.642                      0.642
F000             540.0                      540.0
F000'            541.25
h,k,lmax         11,17,20                   11,17,20
Nref             8082                       8026
Tmin,Tmax        0.891,0.926                0.645,0.746
Tmin'            0.868

Correction method= # Reported T Limits: Tmin=0.645 Tmax=0.746
AbsCorr = MULTI-SCAN

Data completeness= 0.993          Theta(max)= 30.466

R(reflections)= 0.0451( 5804)     wR2(reflections)= 0.1089( 8026)

S = 1.012                  Npar= 291
```

The following ALERTS were generated. Each ALERT has the format
test-name_ALERT_alert-type_alert-level.
Click on the hyperlinks for more details of the test.

Alert level C

```
PLAT911_ALERT_3_C Missing FCF Refl Between Thmin & STh/L=    0.600          3 Report
```

Alert level G

```
PLAT154_ALERT_1_G The s.u.'s on the Cell Angles are Equal ..(Note)      0.002 Degree
PLAT232_ALERT_2_G Hirshfeld Test Diff (M-X)  Mn1      --C15      .        9.6 s.u.
PLAT232_ALERT_2_G Hirshfeld Test Diff (M-X)  Mn1      --C16      .        7.1 s.u.
PLAT303_ALERT_2_G Full Occupancy Atom H1        with # Connections       2.00 Check
PLAT303_ALERT_2_G Full Occupancy Atom H2        with # Connections       2.00 Check
PLAT395_ALERT_2_G Deviating  X-O-Y    Angle From 120 for O3            107.4 Degree
PLAT395_ALERT_2_G Deviating  X-O-Y    Angle From 120 for O4            107.6 Degree
PLAT720_ALERT_4_G Number of Unusual/Non-Standard Labels .........          2 Note
PLAT883_ALERT_1_G No Info/Value for _atom_sites_solution_primary .     Please Do !
PLAT912_ALERT_4_G Missing # of FCF Reflections Above STh/L=  0.600        51 Note
PLAT933_ALERT_2_G Number of OMIT Records in Embedded .res File ...         1 Note
PLAT978_ALERT_2_G Number C-C Bonds with Positive Residual Density.         4 Info
```

```
   0 ALERT level A = Most likely a serious problem - resolve or explain
   0 ALERT level B = A potentially serious problem, consider carefully
   1 ALERT level C = Check. Ensure it is not caused by an omission or oversight
  12 ALERT level G = General information/check it is not something unexpected

   2 ALERT type 1 CIF construction/syntax error, inconsistent or missing data
   8 ALERT type 2 Indicator that the structure model may be wrong or deficient
   1 ALERT type 3 Indicator that the structure quality may be low
   2 ALERT type 4 Improvement, methodology, query or suggestion
   0 ALERT type 5 Informative message, check
```

It is advisable to attempt to resolve as many as possible of the alerts in all categories. Often the minor alerts point to easily fixed oversights, errors and omissions in your CIF or refinement strategy, so attention to these fine details can be worthwhile. In order to resolve some of the more serious problems it may be necessary to carry out additional measurements or structure refinements. However, the purpose of your study may justify the reported deviations and the more serious of these should normally be commented upon in the discussion or experimental section of a paper or in the "special_details" fields of the CIF. checkCIF was carefully designed to identify outliers and unusual parameters, but every test has its limitations and alerts that are not important in a particular case may appear. Conversely, the absence of alerts does not guarantee there are no aspects of the results needing attention. It is up to the individual to critically assess their own results and, if necessary, seek expert advice.

Publication of your CIF in IUCr journals

A basic structural check has been run on your CIF. These basic checks will be run on all CIFs submitted for publication in IUCr journals (*Acta Crystallographica*, *Journal of Applied Crystallography*, *Journal of Synchrotron Radiation*); however, if you intend to submit to *Acta Crystallographica Section C* or *E* or *IUCrData*, you should make sure that full publication checks are run on the final version of your CIF prior to submission.

Publication of your CIF in other journals

Please refer to the *Notes for Authors* of the relevant journal for any special instructions relating to CIF submission.

PLATON version of 13/07/2021; check.def file version of 13/07/2021

Datablock Mndippe_borane - ellipsoid plot

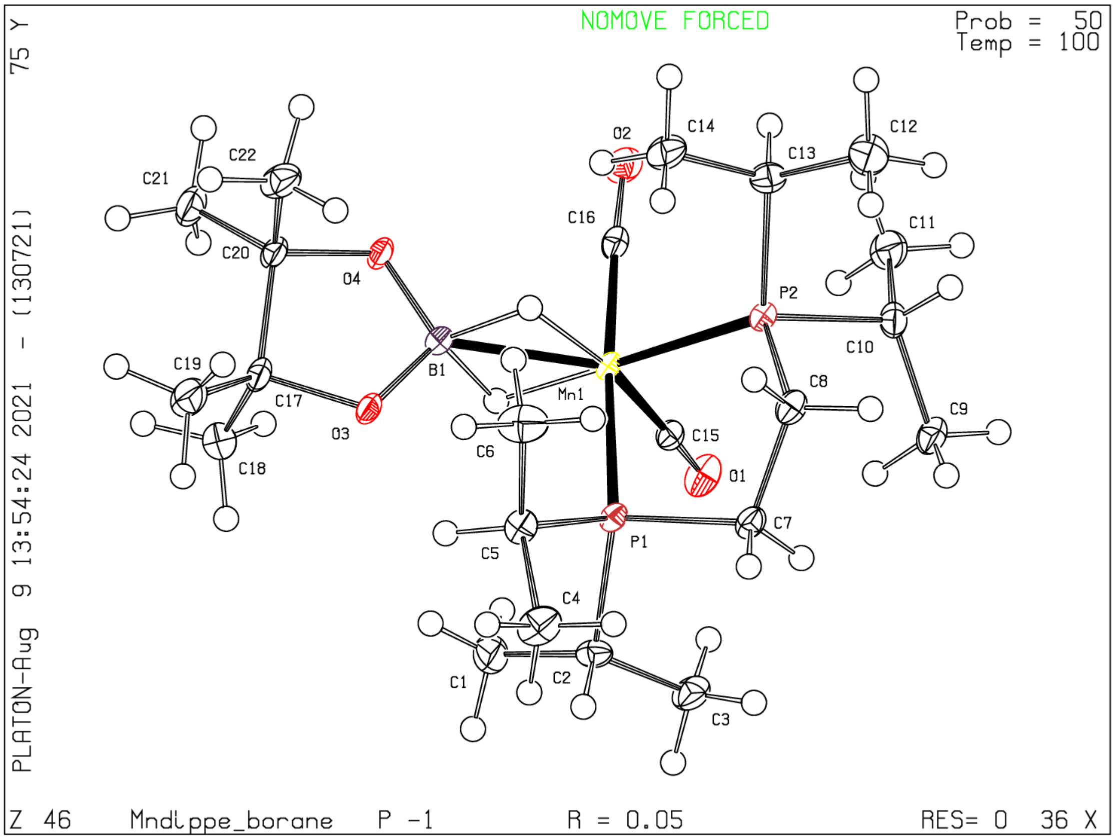

Supplement: Supplementary file 1 — Supporting Information [file ANGE-133-24693-s001.zip › checkcif.pdf]
